# Relationship between Added Sugars Consumption and Chronic Disease Risk Factors: Current Understanding

**DOI:** 10.3390/nu8110697

**Published:** 2016-11-04

**Authors:** James M. Rippe, Theodore J. Angelopoulos

**Affiliations:** 1 Rippe Lifestyle Institute, Quinsigamond Avenue, Shrewsbury, MA 01545, USA; 2 Department of Biomedical Sciences, University of Central Florida, Orlando, FL 32826, USA; 3 School of Health Sciences, Emory & Henry College, Emory, VA 24327, USA; tangelopoulos@ehc.edu

**Keywords:** sucrose, high fructose corn syrup, diabetes, cardiovascular disease, obesity

## Abstract

Added sugars are a controversial and hotly debated topic. Consumption of added sugars has been implicated in increased risk of a variety of chronic diseases including obesity, cardiovascular disease, diabetes and non-alcoholic fatty liver disease (NAFLD) as well as cognitive decline and even some cancers. Support for these putative associations has been challenged, however, on a variety of fronts. The purpose of the current review is to summarize high impact evidence including systematic reviews, meta-analyses, and randomized controlled trials (RCTs), in an attempt to provide an overview of current evidence related to added sugars and health considerations. This paper is an extension of a symposium held at the Experimental Biology 2015 conference entitled “Sweeteners and Health: Current Understandings, Controversies, Recent Research Findings and Directions for Future Research”. We conclude based on high quality evidence from randomized controlled trials (RCT), systematic reviews and meta-analyses of cohort studies that singling out added sugars as unique culprits for metabolically based diseases such as obesity, diabetes and cardiovascular disease appears inconsistent with modern, high quality evidence and is very unlikely to yield health benefits. While it is prudent to consume added sugars in moderation, the reduction of these components of the diet without other reductions of caloric sources seems unlikely to achieve any meaningful benefit.

## 1. Introduction

An ancient Hindu fable tells of six learned blind men who approach an elephant. All are highly esteemed, but all are blind. The first blind man approaches the elephant and happens to bump up against its broad and sturdy side and declares “the elephant is very like a wall!” The second blind man feels the tusk and cries an elephant is “very much like a spear!” The third happens to grab the elephant’s squirming trunk in his hands and boldly declares the elephant is “very like a snake!” The fourth blind man palpates the leg of the elephant and declares “it is clear the elephant is very like a tree!” The fifth blind man who happens to touch the elephant’s ear declares “even the blindest man can tell that the elephant is very like a fan”. The sixth blind man happens to grasp the swinging tail and declares to his comrades the elephant is “very like a rope!”

What then ensues is a long, passionate argument filled with heated dispute amongst these learned men which gets them nowhere. Although each is partly right, none of them has seen the whole picture (while learned, they are blind, after all!). This fable has been utilized in many different eras and many different cultures to recount arguments in areas as diverse as theology and politics. It illustrates the inaccuracy of seeing only a part of a subject and assuming that it is the whole. It is a cautionary tale that even learned men can sometimes be misled by their preconceived notions or only seeing a portion of the whole.

In the complex world of nutrition and particularly in the study of how the foods we eat relate to such chronic conditions as obesity, diabetes and cardiovascular disease (CVD), we are somewhat like the six blind men. Each of us sees a part of the complex puzzle and may assure our colleagues that, in fact, we have solved the entire riddle for how nutrition relates to various disease processes.

The scientific and medical communities have gone down the road of speculating cause and effect without conclusive evidence many times. We blamed salt consumption for contributing to hypertension [1], yet recent evidence suggests that this relationship is far more complex [2,3]. We blamed dietary cholesterol for contributing to heart disease and warned a generation of Americans to avoid eating egg yolks, although that advice has subsequently been found to lack scientific justification [4].

The latest bête noire in nutrition is sweeteners, whether they be nutritive sweeteners, in general, and fructose containing sugars, in particular, or non-nutritive sweeteners (NNS). With the issue of sweeteners, the scientific community faces the problem of trying to offer advice without seeing the totality of the picture, much like the blind men approaching the elephant. It is time to pause and try to see the entire elephant.

This article is based on a symposium conducted at the Experimental Biology Meeting in March 2015, entitled “Sweeteners and Health: Current understandings, controversies, recent research findings and directions for future research”. It is our hope that by providing a broad approach to high level evidence related to nutritive sweeteners, we can begin to get a clearer picture of the entire “elephant” about sweeteners and health rather than concluding that the health effects are due to a single component.

Added sugars are among the most controversial and hotly debated topics in all of nutrition [5,6,7,8,9,10,11,12,13,14,15,16,17,18,19,20,21,22]. Consumption of added sugars has been associated with increased risk of obesity [23,24,25] as well as increased risk factors for cardiovascular disease (CVD) [26], including dyslipidemia [27,28], elevated blood pressure [20,29,30], diabetes [21,31,32], non-alcoholic fatty liver disease [33,34], and even cognitive decline [35] and cancer [36,37]. Data to support these assertions, however, have been challenged consistently. Often these assertions have been based on research trials which provide added sugars in dosages well above those typically found in human consumption (supraphysiological) [12]. Studies comparing pure fructose to pure glucose, neither which is consumed to any appreciable degree in the human diet, have also been extrapolated to human nutrition [38,39]. Although, some trials have compared sucrose to glucose or starch in isocaloric exchange and demonstrated harm with regard to sucrose in insulin/glucose markers and prediabetes/diabetes. Speculation about chronic conditions based on acute data has frequently been employed [40]. Theoretical models, epidemiologic studies which do not establish cause and effect [31,32,41] or data from animal models which can translate poorly to humans particularly in the areas of nutrition, metabolism, and behavior have further clouded the debate [42,43,44,45]. Further controversy has arisen from failure by investigators to clearly acknowledge the limitations of their studies, and misinterpretation or overly simplistic interpretations by media or failure to acknowledge the totality of the evidence often for political reasons or recognition.

A vast amount of literature has been generated, particularly over the past decade, exploring potential linkages between added sugars and various health related conditions. The purpose of this review is to survey some of the modern science, particularly from high quality research trials such as randomized controlled trials, systematic reviews and meta-analyses, in an attempt to provide some clarity in this controversial area. Literature reviews in this manuscript were drawn from articles cited in the World Health Organization report commissioned by Te Morenga et al. [46], articles included in meta-analyses and systematic reviews utilized by the Scientific Advisory Committee on Nutrition (SACN) [47], references utilized by the Dietary Guidelines for Americans 2015–2020 [48], the American Heart Association statement on Carbohydrates and Cardiovascular Disease Risk [49] and randomized controlled trials conducted in the research laboratory of the two authors.

## 2. Levels of Evidence

**Figure 1:**
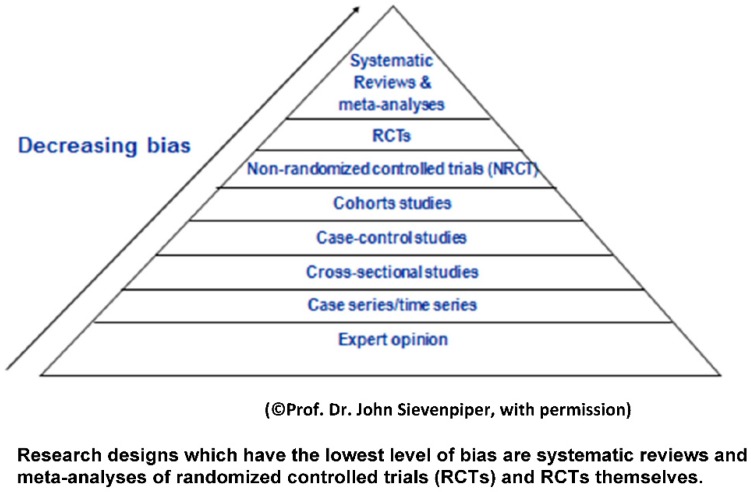
Hierarchy of evidence in evidence based medicine.

Any discussion of health consequences related to added sugars and NNSs must take into account levels of evidence. According to guidelines published both in the United Kingdom and by the US Department of Agriculture (as depicted in Figure 1), the evidence that has the least likelihood of bias is systematic reviews and meta-analyses of randomized controlled trials (RCTs) followed by randomized controlled trials [50]. It should be noted, however, that randomized controlled trials are difficult to apply in the area of nutrition because of the complexity of the field and potential for confounding. Cohort studies (see Table 1) and cross-sectional studies are more prone to bias because of confounding factors that cannot be controlled with this study design. Expert opinion is considered prone to bias as are ecological studies [50].

## 3. Controversies Related to Metabolism of Fructose Containing Sugars

Many of the controversies related to fructose related sugars are based on the well-known differences between metabolism of fructose and glucose in the liver [62]. Over 90% of fructose ingested is absorbed through the small intestine and metabolized in the liver on first pass. In contrast, glucose is metabolized by a variety of organs. It is important to note, however, that the pathways are interactive. Numerous studies including isotope studies have shown that roughly 50% of fructose is converted to glucose within the liver. An additional 15%–20% is converted to glycogen, 20%–25% to lactate, and a few percent to carbon dioxide [62,63]. Multiple studies have shown that only 1%–5% of consumed fructose may follow the pathway of de novo lipogenesis and be converted into free fatty acids which are then packaged as triglycerides and either stored in the liver or released in the bloodstream [62,64,65]. Some short-term data with very large doses of pure fructose have suggested that increases in liver fat can be achieved over a short period of time; Faeh et al. gave seven healthy men six days of a high fructose diet comprising an extra 25% of total calories and demonstrated suppression of adipose tissue lipolysis [66].

Schwarz et al. utilizing a diet with 25% pure fructose demonstrated increased fractional hepatic DNL and liver fat [67]. Schwarz et al. studied 25 Latino children and 15 African American children and demonstrated over a ten-day period that replacing high fructose products with vegetables, bread or pasta demonstrated decreased liver fat in this population [68].

In certain animals, de novo lipogenesis can be a major pathway [69]. In humans, it is minimal. Some investigators have misinterpreted the effect of this pathway in humans to contend that fructose consumption can result in increased risk of non-alcoholic fatty liver disease (NAFLD) and insulin resistance [15].

The modern challenge to fructose, in retrospect, came from an opinion piece published in 2004 in the American Journal of Clinical Nutrition by Bray, Nielson and Popkin which asserted that “the increase in consumption of HFCS has a temporal relation to the epidemic of obesity, and the overconsumption of HFCS in calorically sweetened beverages may play a role in the epidemic of obesity” [8]. The authors were careful to point out that this temporal association did not establish cause and effect. It was widely misinterpreted by other scientists and the public at large to suggest that there was something unique about HFCS related to obesity. Subsequent research has shown that HFCS and sucrose have indistinguishable metabolic effects and health consequences in human beings [70,71,72].

It is also worth noting that sugar consumption has declined significantly in the United States, Britain, Canada, and Australia at a time when obesity rates have continued to rise. This was first reported in Australia and has become known as the “Australian Paradox” [73]. Furthermore, Mozaffarian et al. reported the impact of increased servings of different food and weight change over a four-year interval by combining Nurses’ Health Study (NHSI) (1986–2006), NHSII (1991–2003), and the Health Professionals Follow-up Study (1986–2006) for a combined cohort of a 120,877 people. After multivariable-adjustment for age, Body Mass Index (BMI), sleep, physical activity, alcohol, television watching, smoking and all other dietary factors (French fries, potato chips, processed meat and red meats) all resulted in more weight gain over each four year period than did sugar sweetened beverages (SSB) [74]. These data should be treated with some caution since they come from cohort studies and do not represent a randomized controlled trial. It may be that all of these food products are simply markers for an overall diet that is energy dense and that it is the overall diet pattern, and not any individual component of it, that is associated with weight gain.

## 4. Effects of Sugars on Body Weight and Body Composition

It has been argued that consumption of sugars may predispose individuals to increase in adiposity, weight gain and ultimately overweight and obesity. A number of randomized controlled trials (RCT) have been performed exploring sugar consumption and weight. These RCTs have been aggregated in four recent meta-analyses, however, these studies employ different inclusion and exclusion criteria and reported different summary endpoint estimates and conclusions [46,75,76,77] (See Table 2). Sievenpiper et al. [76] and Te Morenga et al. [46] looked at isocaloric exchange of either sugar or fructose with other macronutrients to assess effect of body weight in adults. Neither of these analyses showed significant effect of either sugar or fructose on body weight. With regard to sugars and weight loss Te Morenga et al. reviewed RCTs to examine whether or not the effect of weight and calories from sugars are reduced [46]. These investigators performed meta-analyses on five trials in children and demonstrated no significance in isocaloric trials of children and adults. A meta-analysis by Malik et al. found two of five trials resulted in significant weight loss resulting from a reduction in sugar calories in one model but not another [77]. It should be pointed out that in the trials that were meta-analyzed, subjects consumed not only less calories from sugar, but less total energy. Thus, it is not clear that the weight loss resulted from reduction in calories from sugar.

These four research groups also conducted meta-analyses in studies where an increased amount of sugar calories was given to adults who were consuming ad libitum diets. All four meta-analyses reported significant weight gain in this model although individual studies often did not. Thus, it is not clear whether the change in weight was due to an increase in the total number of calories consumed or some unique property of sugars. Recent meta-analyses by Dolan et al. of interventional studies utilizing the FDA Guidance for Evidence-Based Review both in normal weight [78] and obese individuals [79] did not support a link between obesity and fructose consumption with amounts up to the 90th percentile population consumption for fructose.

The report of the SACN in the UK, which is based on an extensive series of systematic reviews conducted according to clearly stated quality standards, reported that high levels of free sugar consumption were associated with excess energy intake [47]. Thus, weight gain in these studies could not be separated from calorie intake and could not be attributed to any unique property of free sugars. Although it could be argued that free sugar consumption may predispose to excess calorie intake. It has also been reported that fructose containing sugars may predispose individuals to abdominal weight gain [80,81]. If this were true, it would represent a significant increased risk for both diabetes and the metabolic syndrome. Stanhope et al. reported a research trial comparing 25% of calories from fructose to 25% of calories from glucose [81]. Individuals in the fructose arm, over a 10-week period, increased their visceral abdominal fat. However, it should be noted that individuals also gained an average of two pounds over the course of this study. Furthermore, significance in abdominal weight gain occurred only pre-to-post in the fructose arm and this was not compared to the glucose arm. When this more appropriate glucose to fructose comparison was made, the significance disappeared. Maersk et al. [80] conducted a six-month study comparing one liter per day of sugar sweetened beverage versus comparable amounts of diet beverage, 1% milk, and water. These investigators reported that individuals in the sugar sweetened beverage group increased visceral abdominal fat compared to the other groups. It should be noted, however, that individuals also gained weight in this study which represents a confounding variable.

Three recent RCTs have been conducted employing slightly different strategies have explored aspects of sugar consumption and weight change. In one study, consumption of average amounts of fructose containing sugars for adults (HFCS or sucrose) did not result in increased body weight over a ten-week, free living trial [51]. In another study, mean amounts of these sugars were utilized as part of an overall hypocaloric diet and did not inhibit weight loss [52]. Of note, there were no differences between 10% and 20% of either HFCS or sucrose. In a larger trial involving 355 men and women who consumed either 8%, 18% or 30% of kcals/day of either sucrose or HFCS as part of a mixed nutrient diet, individuals gained an average of slightly over two pounds over a ten-week period. However, most of this was driven by the 30% kcals per day (above the 95% population consumption for fructose) [53]. At the end of the study, individuals consumed an average of more than 200 kcals/day compared to baseline. Thus, this should be viewed as a hypercaloric trial.

Fructose containing sugars led to the expected weight loss (with some exceptions in children) in subtraction trials which suggests that fructose containing sugars do not behave differently from other macronutrients (mainly starch) when comparisons are matched for calories. Another approach to this issue may be obtained from an *ad libitum* trial design where fructose containing sugars were freely replaced with other sources of energy in the diet and no strict control of the amount of sugars in the background diet occurred. CArbohydrate Ratio Management in European National Diets (CARMEN) trial [84] is the largest and longest trial using such a design. This diet compared ad libitum high complex carbohydrate diet to an ad libitum higher fat control trial in 398 obese individuals studied for over six months. Both ad libitum diets resulted in lost weight. There was no significant different between the ad libitum high sugars diet and the ad libitum high complex carbohydrate diet. There was a non-significant tendency toward greater weight loss in the latter. This trial also showed that under free living conditions it is possible to lose weight following an ad libitum high sugars diet employing a strategy to freely replace energy from high fructose containing sugars with other sources of energy in the diet. It also demonstrates that there is not clear advantage for reducing sugars as compared to fat in the diet [46,75,76,77]. Given the complexity of weight gain and energy regulation it is unlikely that one component of the diet significantly impacts upon this problem. In fact, the consensus statement from the American Society of Nutrition on energy regulation specifically warns against isolating one component of the diet and blaming it for obesity [85]. Moreover, a large body of literature associates both increased caloric consumption from all sources [86] and decreased physical activity [87] as major components of weight gain. Indeed, the average American consumed 454 more calories in 2010 compared with 1970. Of these additional calories, 93% came from increased consumption of flour and cereal products or fats while only 7% (39 additional calories) came from all sugars combined. The percentage of calories from sugar in the diet in the United States actually declined from 19% to 17% over this period [88]. It should be pointed out, however, that sugars may provide excess energy due to their hedonic properties. In addition, increased sugars intake in some individuals may be a marker for an overall less healthy, energy dense diet.

The recent literature on the impact of added sugars on obesity and weight gain or weight loss remains in dispute. Most of the RCTs suggest that weight gain occurs only in hypercaloric trials and suggests that overall caloric consumption is likely to be a larger contributor to weight gain than any unique property of sugars [74,75].

## 5. Risk Factors for Diabetes

Considerable confusion exists with regard to the potential impact of added sugars on risk factors for diabetes. A great deal of attention was paid to this issue in the media following two ecological studies which suggested that availability of sugars correlated with increased risk of diabetes [31,32]. These types of ecological studies, however, must be treated with great caution. Ecological studies are considered one of the lowest forms of evidence. Furthermore, these studies have been criticized on a variety of technical grounds. In one ecological study, Goran et al. [32] reported that diabetes prevalence was 20% higher in European Union (EU) countries with higher availability of HFCS compared to countries with low availability. As noted by van Buul et al. however, HFCS consumption data in EU countries reported in this study were, in fact, not consumption data at all but production data [5]. Since HFCS travels freely across EU borders, production data cannot be assumed to be the equivalent of consumption data. In another ecological study, Basu et al. used food supply data from the UNFAO to determine market availability of different food items worldwide and concluded that sugar availability was associated with higher diabetes prevalence. Market availability of food, however, is a highly unreliable indicator of sugar consumption [6].

Prospective cohort studies have not documented a direct relationship between fructose and diabetes [89]. Pooled analysis of these cohorts did reveal that SSBs as a source of free sugar are associated with an increased risk of diabetes only when comparing highest and lowest levels of exposure [22,90]. Pooled analyses of these cohorts, however, for total sugars, total sucrose, and total fructose have not yielded the same relationship [91]. In addition, systematic reviews and meta-analyses of sugar and diabetes risk factors have actually reported a decrease in risk factors such as glycosylated proteins [82]. A large cohort study in Europe also did not show an increase in diabetes risk with added sugars [92].

The question of whether or not sugar is a unique cause of diabetes has not been addressed in any RCT to our knowledge. Most of the data related to the question of a potential relationship between sugar consumption and diabetes comes from RCTs looking at risk factors for diabetes or cohort studies. Prospective cohort studies provide mixed evidence concerning sugar consumption and diabetes. Malik et al. reported meta-analyses of eight cohort studies, four of which did not find a significant effect of SSB with the incidence of diabetes and five did not adjust findings for energy intake and body weight [22]. A study published by the same group did not show a relation between sugar consumption and the risk of diabetes [93]. Other cohort studies have also failed to find significant associations between sugar intake and diabetes [94,95,96] and one study found a significant negative association [95]. With regard to systematic reviews and meta-analyses, few data are available to support an association between sugar intake and diabetes [94,95,96]. Cozma et al. reported a systematic review and meta-analysis of 18 feeding studies on fructose and diabetes risk and found no adverse impact on glycemic control including insulin, glucose, glycated blood proteins (including HbA1c) [82]. The SACN report published in 2015 [47] did not show an association between free sugars consumption and risk factors for diabetes.

Most randomized controlled trials of non-diabetic patients substituting sucrose for fructose in a controlled diet did not report adverse effects on multiple risk factors for diabetes [70,78,97,98,99].

Two recent RCTs have also not demonstrated increased risk factors for diabetes over a 10-week time period. In one study of 123 individuals who consumed average levels of fructose containing sugars (9% of calories from fructose itself or 18% of calories from either sucrose or HFCS) did not yield increases in fasting glucose, insulin, or insulin resistance via the homeostatic model of assessment (HOMA) [100]. Another RCT evaluated 267 individuals who consumed either HFCS or sucrose at dosage ranges between 8% and 30% of calories (25th through 95th percentile of calories) and also did not find any increase in risk factors for diabetes [53].

This literature taken together provides little direct evidence that sugar consumption increases risk factors of diabetes. Moreover, since the relationship between diabetes and obesity is well established and, as already indicated, scant evidence is available relating isocaloric substitution of sugars for other carbohydrates, it appears prudent to focus on other risk factors for diabetes such as obesity rather than singling out sugars. Since diabetes takes 20–30 years to develop short-term RCTs focusing on risk factors for diabetes should be taken with caution recognizing this limitation.

## 6. Risk Factors for Cardiovascular Disease

The American Heart Association (AHA) has recommended that adult males consume no more than 150 kcals per day and females no more than 100 kcals per day from added sugars [101]. This recommendation implies that higher levels of added sugars may increase the risk of heart disease. In addition, the DGAC 2015 concluded that there was “moderate” evidence in the association between added sugars and heart disease [48]. The SACN report published in 2015 did not find a linkage between sugars consumption and risk factors for heart disease [47]. The evidence in this area, however, is mixed and inconclusive [13]. To our knowledge there are no RCTs assessing a link between added sugars and CVD. Thus, the available data comes either from cohort studies or from RCTs examining risk factors for CVD.

Dietary sugars may have differential effects on blood lipids. A number of studies have demonstrated that diets containing greater than 20% of kcals from simple sugars may result in elevated fasting triglycerides which is a known risk factor for CVD (see Table 3) [32,54,55,56,57,58,59,60,61,99]. The American Heart Association Scientific Statement on triglycerides lists avoiding excess fructose as one mechanism for decreasing the risk of hypertriglyceridemia [102]. Several recent systematic reviews and meta-analyses, however, have reported that in trials where fructose is substituted isocalorically for other carbohydrates it does not result in increased fasting triglycerides or post-prandial triglycerides [103,104].

Two recent RCTs looked at the relationship between sugar consumption and triglycerides. In one involving 65 individuals where no weight gain occurred, no increase in triglycerides was found [51]. A larger trial involving 355 men and women who consumed between 8% and 30% of kcals per day as either sucrose or HFCS as part of a mixed nutrient diet reported a 10% increase in triglycerides [53]. It should be pointed out, however, that individuals in this trial gained approximately two pounds over the ten-week intervention and were consuming an average of over 200 kcals per day, more by the end of the study compared to baseline. Stanhope et al. followed various doses of HFCS given to young adults over a 16-day period and also reported increases in post-prandial triglycerides [107]. However, the short duration of this study and the fact that pre and post levels were within the low normal range must be taken into consideration when evaluating this finding.

The effects of added sugars on low density lipoprotein (LDL) have been variable [27,59,80,102,108] with some investigators reporting increases while other studies have not demonstrated this finding. It should be noted that a number of the trials where the increases in LDL occurred gave large dosages of added sugars often above the 90th percentile population [109].

A study by Yang et al. published in 2013 analyzed NHANES data from three different time periods (1988–1984, 1999–2004 and 2005–2010) and reported that the relative risk was 1.30 for those who consumed 10%–24.9% of calories from added sugars and 2.75 for those who consumed 25% or more calories from added sugars (approximately 10% of the population) when compared to those who consumed less than 10% of calories from added sugars. It should be noted that the authors also reported that the percentage of daily calories from added sugars was 16.8% in the 1999–2004 cohort and decreased to 14.9% in the 2005–2010 cohort [106]. Several RCTs involving levels of sugar consumption ranging from the 25th to the 95th percentile population consumption have demonstrated no changes in LDL cholesterol following ten weeks in a free living environment compared to baseline when consumed as part of mixed nutrient diet [53]. Thus, the effects of added sugars on lipids in adults remain in dispute.

Research evaluating the effects of added sugars on blood pressure have similarly shown mixed results [29,30,110]. For example, epidemiologic studies such as the Framingham Heart Study have reported an association between consuming one or more SSB per day and increased odds of developing high blood pressure [111]. The meta-analysis by Te Morenga et al. which reported on 12 trials (*n* = 324) found no significant effects of higher sugar intake on systolic blood pressure overall, although higher sugar intake was associated with significant increase in diastolic pressure of 1.4 mm/hg (95% CI: 0.3, 2.5 mm/hg; *p* = 0.02) [109]. Many of the trials reported in this systematic review, however, employed amounts of added sugars consumption above the 90th percentile population consumption level. A systematic review and meta-analysis by Ha and colleagues, involving 18 studies (*n* = 355), showed slight decreases in both diastolic and mean blood pressure when fructose was substituted either isocalorically for other carbohydrates (13 trials) or in hypercaloric trials (2 trials) [83]. Several recent RCTs have not shown increases in blood pressure. In a large study of 355 individuals followed for ten weeks at up to 30% of kcals per day up to the 95% percentile population consumption level of fructose [53], no increases in blood pressure were observed. Further RCTs compared fructose containing sugars to glucose at the 50th percentile population consumption and did not demonstrate increases in mean systolic or diastolic blood pressure [51].

Thus, if there is any association between sugar consumption and increases in blood pressure it would appear to occur at higher levels of sugar consumption (>90th percentile population consumption) and even at that level may not exist.

Taken as a whole, it does not appear that sugar consumption within the normal range of the human diet increases the risk of cardiovascular disease. An exception, however, may occur with diets that contain greater than 20% of kcals from simple sugars in hypercaloric trials which may cause an increase in triglycerides. It should be noted that Archer et al. utilized NHANES data (NHANES 1988–1994, 1999–2004 and 2015) (*n* = 31,147) compared to the NHANES III Mortality Report (1988–2006) (*n* = 11,733) and reported that individuals who consumed 25% or more of calories from added sugars (approximately 77% of the population) experienced an increased associated risk of cardiovascular disease [105] compared to those who consumed less than 10% of calories from added sugars. These findings should be treated with caution given the multiple potential confounders inherent to all cohort studies. In particular, NHANES data has recently been challenged because of its use of memory based recall which has been found in multiple studies to be highly inaccurate. These investigators also noted that the percentage of daily calories from added sugars declined from 1999 to 2004 with a decline from 16.8% to 14.9% in 2005–2010 (9% decline).

To put the issue of SSB consumption in perspective, it should be noted that the major risk factors for heart disease are well established such as avoiding cigarette smoking, maintaining a proper weight, avoiding or controlling diabetes and leading a physically active lifestyle. It would appear prudent to focus more attention on these established risk factors than one component of overall approach to nutrition. RCTs of longer duration would be helpful in examining putative links between sugar consumption and risk factors for CVD.

## 7. Effects of Sugars on the Brain

The effects of sugar on the brain, in general, and on reward pathways, in particular, as well as on downstream portions of the brain has been an area of intense research and controversy. Early studies in this area were done largely on animals [43,112,113,114], however, recent advances in functional MRI (fMRI) have allowed more studies to be conducted in human beings [115]. Animal studies in this area must be treated with great caution since there are multiple and significant differences between animal brains (in particular, rodents which are the most frequently used model) and human brains [116,117]. Further confusion in this area has come from studies which have utilized a model comparing fructose versus glucose to examine effects on blood flow to the hypothalamus and reward pathways despite the fact that these monosaccharides are rarely consumed by themselves in human nutrition [118,119]. Unfortunately, these trials of two monosaccharides in isolation have led to speculation that fructose and glucose interact differently in the brain thereby leading to potential for overconsumption of calories.

When similar studies have been repeated comparing the normally consumed sugars of sucrose or HFCS on blood flow to the hypothalamus and brain connectively, no differences have been reported between sweetened beverages consumed in the context of a mixed nutrient meal and an unsweetened control [120].

Stice et al. reported a trial of 70 individuals comparing various levels of sugar sweetened milkshakes to various levels of fat in milkshakes and reported that there was more stimulation of reward pathways following the highest level of sugar than fat [40]. These investigators speculated that these acute findings suggested that sugar should be regulated rather than fat with regard to lowering the prevalence of obesity. There are studies, however, which show exactly the opposite [121,122].

Stephan et al. [35] using epidemiologic data suggested that increased consumption of fructose containing sugars could lead to dementia. Studies performed ranging in duration from 10 weeks to 24 weeks and employing average levels of consumption of fructose containing sugars have not found any evidence of cognitive change [123,124].

Unfortunately, some investigators have speculated that sweetness from added sugars may lead to a form of sugar “addiction” [15,125]. Animal data has also been used to buttress this claim [126,127] despite the fact that the translation of animal data to humans in this area is fraught with complexity and speculation. Several recent reviews have provided extensive analyses questioning the fundamental premise of either food or sugar “addiction” [128,129,130]. Unfortunately, the popular press and the public has embraced the concept of sugar “addiction” which would appear to be a vast exaggeration of what the scientific data show. Clearly, this is an area where much more research is required.

## 8. Conclusions

There is no question that multiple, important links exist between nutrition and health. The current emphasis on added sugars, however, has created an environment that is “sugar centric” and in our judgment risks exaggerating the effects of these components of the diet with the potential unforeseen side effect of ignoring other important nutritional practices where significant evidence of linkages to health exists.

We have seen the attempt to focus on single nutrients in the diet before attempting to blame a variety of chronic illnesses on overconsumption of these components of the diet [131]. For example, dietary cholesterol was initially blamed as a significant positive factor in coronary artery disease although subsequent research has not supported this linkage. Subsequently, saturated fats were deemed to be a villain although recent evidence now suggests that the food matrix containing the saturated fats may be more important than the saturated fats themselves with regard to risk of CVD [132,133,134].

The same phenomenon may hold true for isolating components of the diet for supposed health benefits [135]. For example, even though oats have multiple health benefits, the exaggerated health claims caused one pundit to suggest that putting oats in carbonated soft drinks could lead to increase in their sales. There are multiple benefits of consuming protein yet the current fashion of critically accepting high protein diets for a variety of potential health benefits seems overwrought. These are but two of many examples. One has only to look at the popular press to find the current month’s super food.

The history of nutrition is littered with attempts to isolate one nutrient, or class of nutrients, to claim a plethora of benefits or risk [131]. These have almost universally resulted in failure and disappointment. In the area of sugar sweetened beverages and various health considerations, the highest quality of evidence from systematic reviews, meta-analyses, and randomized controlled trials does not suggest signals for harm within the normal range of human consumption at least in short-term studies lasting six months or less and in longer-term cohort studies where fructose containing sugars are substituted isocalorically for other carbohydrates. This would suggest that some of the recently articulated restrictive guidelines from prestigious scientific and health organizations may be overly restrictive although longer term studies will be required to provide more certainty on this issue.

We wish to emphasize that we are not recommending excessive consumption of added sugars. It would appear to the authors, however, that a reasonable recommended upper limit of sugar may reside at consuming no more than 20% of calories from added sugars and then only in a hypercaloric situation. This recommendation rests largely on our view that the evidence suggests a potential signal for elevated triglycerides at consumption levels greater than 20% of calories in hypercaloric trials. We recognize, however, that definitive evidence in this area may be very difficult to generate. Longer term RCTs, particularly, with ad libitum sugar consumption designs may prove helpful. Current ad libitum trials are typically of a short duration.

There are well established risk factors for obesity, diabetes, and cardiovascular disease and considerable overlap amongst these entities when it comes to nutritional practices. For now, we would agree with the assertion in the Dietary Guidelines for American (2010) [136] that overconsumption of calories represents the single greatest health threat to individuals in the United States and elsewhere. This may, in part, be linked to the overall consumption patterns in what has been called the “Western” diet. Certainly, added sugars may be considered as components of this overall diet and, therefore, targets for reduction as are other energy dense components of this nutrition pattern. Singling out added sugars as major or unique culprits for metabolically based diseases such as obesity, diabetes, and cardiovascular disease appears inconsistent with modern high quality evidence and is very unlikely to yield health benefits. The reduction of these components of the diet without other reductions seems very unlikely to achieve any meaningful results. Perhaps in this situation, we should remember a favorite quotation of President John F. Kennedy who quoted Winston Churchill who, in turn, had paraphrased the philosopher George Santayana by saying “Those who fail to learn from history are doomed to repeat it”.

## Figures and Tables

**Table 1:** Randomized Control Trials Included.

	Type of Analysis	Findings
Lowndes et al. [51]	50th percentile consumption of fructose containing sugars	No increase in body weight over 10 weeks and no increase in triglycerides. No increase in risk factors for diabetes
Lowndes et al. [52]	Comparison between 10 and 20 percent of calories from either HFCS or sucrose in hypocaloric diets	Significant weight loss occurred in all groups
Lowndes et al. [53]	RCT 355 men and women consuming 8%, 18% or 30% of kcals per days either sucrose or HFCS	Average weight gain over 2 pounds over 10 week period. Mostly driven by 30% kcal per day group. No increased risk factors for diabetes. 10% increase in triglycerides confounded by 2 pound weight gain.
Antar et al. [54]	Randomized Control Trial	Increase in fasting triglycerides from various levels of added sugar consumption
Bantle et al. [55]	Randomized Control Trial	Increase in fasting triglycerides from various levels of added sugar consumption
Black et al. [56]	Randomized Control Trial	Increase in fasting triglycerides from various levels of added sugar consumption
Cooper et al. [57]	Randomized Control Trial	Increase in fasting triglycerides from various levels of added sugar consumption
Groen et al. [58]	Randomized Control Trial	Increase in fasting triglycerides from various levels of added sugar consumption
Marckmann et al. [59]	Randomized Control Trial	Increase in fasting triglycerides from various levels of added sugar consumption
Sorensen et al. [60]	Randomized Control Trial	Increase in fasting triglycerides from various levels of added sugar consumption
Stanhope et al. [61]	Randomized Control Trial	Increase in fasting triglycerides from various levels of added sugar consumption

**Table 2:** Systematic Reviews and Meta-analyses Included.

	Type of Analysis	Findings
Sievenpiper et al. [76]	Aggregated randomized control trials looking at isocaloric exchange of either sugar or fructose with other macronutrients to assess effects on body weight in adults	No significant effect of either sugar or fructose on body weight
Te Morenga et al. [46]	Aggregated randomized control trials looking at isocaloric exchange of either sugar or fructose with other macronutrients to assess effects on body weight in adults	No significant effect of either sugar or fructose on body weight
Malik et al. [77]	Meta-analysis of 5 trials	2 of 5 trials resulted in significant weight loss from reducing sugar calories in one model but not another
Dolan et al. [78]	Normal weight individuals. Interventional Studies utilizing the FDA guidance for evidence based reviews	No difference with regard to obesity from fructose consumption in normal weight individuals
Dolan et al. [79]	Obese individuals. Interventional Studies utilizing the FDA guidance for evidence based reviews	No difference with regard to obesity from fructose consumption in obese individuals
Cozma et al. [82]	Systematic review and meta-analysis of 18 RCTs	Decrease in risk factors for diabetes such as glycosylated proteins
Malik et al. [24]	Meta-analysis of 8 cohort studies	4 did not find a significant effect of SSB on incidence of diabetes and 5 did not adjust findings for energy intake and body weight
Ha et al. [83]	15 studies involving 355 individuals	Slight decreases in diastolic and mean blood pressure and isocaloric substitution or hypercaloric trials

**Table 3:** Cohort Studies Included.

	Type of Analysis	Findings
Hodge et al. [94]	Cohort Study	No significant association between sugar intake and diabetes
Meyer et al. [95]	Cohort Study in Older women	Significant negative association between sugar intake and diabetes
Colditz et al. [96]	Cohort Study in women	No association between sugar intake and diabetes
Interact [92]	Cohort Study in European Adults	No increase in diabetes risk with added sugars
Archer et al. [105]	NHANES data analysis	Individuals who consumed 25% or more of calories from added sugars experienced an increase associated risk of cardiovascular disease compared to individuals who consumed less than 10% of calories from added sugars
Yang et al. [106]	NHANES data analysis	CVD risk increased to 1.30 for individuals who consumed 10 to 24.9% of calories and 2.75 for those who consumed 25% or more calories for added sugars compared to individuals who consumed less than 10% of calories from added sugars

## Abbreviations

The following abbreviations are used in this manuscript:

AHA	American Heart Association
CVD	Cardiovascular Disease
fMRI	Functional MRI
HOMA	Homeostatic Model of Assessment
LDL	Low Density Lipoprotein
NAFLD	Non-Alcoholic Fatty Liver Disease
RCTs	Randomized Controlled Trials
SSB	Sugar Sweetened Beverages
NNS	Non-Nutritive Sweeteners
